# Supplementary material for: HIVprotI: an integrated web based platform for prediction and design of HIV proteins inhibitors
Source: J Cheminform. 2018 Mar 9;10:12. doi: 10.1186/s13321-018-0266-y (PMC5845081; doi:10.1186/s13321-018-0266-y)
Supplement: Supplementary file 2 — Additional file 2. Source code of HIVProtI web server. [file 13321_2018_266_MOESM2_ESM.zip › HIVprotI_Source-code/hivprotI/weka-3-6-6/doc/weka/clusterers/forOPTICSAndDBScan/Databases/Database.html]

Database


---


|  |  |  |
| --- | --- | --- |
| **Overview** \| **Package** \| **Class** \| **Tree** \| **Deprecated** \| **Index** \| **Help** \| **Weka's home** | |  |
| PREV CLASS   **NEXT CLASS** | **FRAMES**    **NO FRAMES**     **All Classes** |  |
| SUMMARY: NESTED \| FIELD \| CONSTR \| METHOD | DETAIL: FIELD \| CONSTR \| METHOD |  |


---


## weka.clusterers.forOPTICSAndDBScan.Databases Interface Database

**All Known Implementing Classes:**: SequentialDatabase

---

``` public interface Database ```

Database.java

**Version:**
:   $Revision: 1.2 $

**Author:**
:   Matthias Schubert (schubert@dbs.ifi.lmu.de), Zhanna Melnikova-Albrecht (melnikov@cip.ifi.lmu.de), Rainer Holzmann (holzmann@cip.ifi.lmu.de)

---

| **Method Summary** | |
| --- | --- |
| `boolean` | `contains(DataObject dataObject_Query)`             Tests if the database contains the dataObject\_Query |
| `java.util.List` | `coreDistance(int minPoints, double epsilon, DataObject dataObject)`             Calculates the coreDistance for the specified DataObject. |
| `java.util.Iterator` | `dataObjectIterator()`             Returns an iterator over all the dataObjects in the database |
| `java.util.List` | `epsilonRangeQuery(double epsilon, DataObject queryDataObject)`             Performs an epsilon range query for this dataObject |
| `double[]` | `getAttributeMaxValues()`             Returns the array of maximum-values for each attribute |
| `double[]` | `getAttributeMinValues()`             Returns the array of minimum-values for each attribute |
| `DataObject` | `getDataObject(java.lang.String key)`             Select a dataObject from the database |
| `Instances` | `getInstances()`             Returns the original instances delivered from WEKA |
| `void` | `insert(DataObject dataObject)`             Inserts a new dataObject into the database |
| `java.util.List` | `k_nextNeighbourQuery(int k, double epsilon, DataObject dataObject)`             Emits the k next-neighbours and performs an epsilon-range-query at the parallel. |
| `java.util.Iterator` | `keyIterator()`             Returns an iterator over all the keys |
| `void` | `setMinMaxValues()`             Sets the minimum and maximum values for each attribute in different arrays by walking through every DataObject of the database |
| `int` | `size()`             Returns the size of the database (the number of dataObjects in the database) |

**Method Detail**

### getDataObject

```
DataObject getDataObject(java.lang.String key)
```

:   Select a dataObject from the database

    :   **Parameters:**: `key` - The key that is associated with the dataObject **Returns:**: dataObject

---


### size

```
int size()
```

:   Returns the size of the database (the number of dataObjects in the database)

    :   **Returns:**: size

---


### keyIterator

```
java.util.Iterator keyIterator()
```

:   Returns an iterator over all the keys

    :   **Returns:**: iterator

---


### dataObjectIterator

```
java.util.Iterator dataObjectIterator()
```

:   Returns an iterator over all the dataObjects in the database

    :   **Returns:**: iterator

---


### contains

```
boolean contains(DataObject dataObject_Query)
```

:   Tests if the database contains the dataObject\_Query

    :   **Parameters:**: `dataObject_Query` - The query-object **Returns:**: true if the database contains dataObject\_Query, else false

---


### insert

```
void insert(DataObject dataObject)
```

:   Inserts a new dataObject into the database

    :   **Parameters:**: `dataObject` -

---


### getInstances

```
Instances getInstances()
```

:   Returns the original instances delivered from WEKA

    :   **Returns:**: instances

---


### setMinMaxValues

```
void setMinMaxValues()
```

:   Sets the minimum and maximum values for each attribute in different arrays
    by walking through every DataObject of the database

---


### getAttributeMinValues

```
double[] getAttributeMinValues()
```

:   Returns the array of minimum-values for each attribute

    :   **Returns:**: attributeMinValues

---


### getAttributeMaxValues

```
double[] getAttributeMaxValues()
```

:   Returns the array of maximum-values for each attribute

    :   **Returns:**: attributeMaxValues

---


### epsilonRangeQuery

```
java.util.List epsilonRangeQuery(double epsilon,
                                 DataObject queryDataObject)
```

:   Performs an epsilon range query for this dataObject

    :   **Parameters:**: `epsilon` - Specifies the range for the query: `queryDataObject` - The dataObject that is used as query-object for epsilon range query **Returns:**: List with all the DataObjects that are within the specified range

---


### k\_nextNeighbourQuery

```
java.util.List k_nextNeighbourQuery(int k,
                                    double epsilon,
                                    DataObject dataObject)
```

:   Emits the k next-neighbours and performs an epsilon-range-query at the parallel.
    The returned list contains two elements:
    At index=0 --> list with all k next-neighbours;
    At index=1 --> list with all dataObjects within epsilon;

    :   **Parameters:**: `k` - number of next neighbours: `epsilon` - Specifies the range for the query: `dataObject` - the start object **Returns:**: list with the k-next neighbours (PriorityQueueElements) and a list with candidates from the epsilon-range-query (EpsilonRange\_ListElements)

---


### coreDistance

```
java.util.List coreDistance(int minPoints,
                            double epsilon,
                            DataObject dataObject)
```

:   Calculates the coreDistance for the specified DataObject.
    The returned list contains three elements:
    At index=0 --> list with all k next-neighbours;
    At index=1 --> list with all dataObjects within epsilon;
    At index=2 --> coreDistance as Double-value

    :   **Parameters:**: `minPoints` - minPoints-many neighbours within epsilon must be found to have a non-undefined coreDistance: `epsilon` - Specifies the range for the query: `dataObject` - Calculate coreDistance for this dataObject **Returns:**: list with the k-next neighbours (PriorityQueueElements) and a list with candidates from the epsilon-range-query (EpsilonRange\_ListElements) and the double-value for the calculated coreDistance


---


|  |  |  |
| --- | --- | --- |
| **Overview** \| **Package** \| **Class** \| **Tree** \| **Deprecated** \| **Index** \| **Help** \| **Weka's home** | |  |
| PREV CLASS   **NEXT CLASS** | **FRAMES**    **NO FRAMES**     **All Classes** |  |
| SUMMARY: NESTED \| FIELD \| CONSTR \| METHOD | DETAIL: FIELD \| CONSTR \| METHOD |  |


---
